# First evidence of wulfenite in Calabria Region (Southern Italy)

**DOI:** 10.1016/j.dib.2018.05.110

**Published:** 2018-05-23

**Authors:** A. Bloise, L. Dattola, I. Allegretta, R. Terzano, M. Taranto, D. Miriello

**Affiliations:** a Department of Biology, Ecology and Earth Sciences, University of Calabria, Via Pietro Bucci, I-87036 Rende, CS, Italy; b Regional Agency for the Protection of the Environment of the Calabria region (Arpacal), Italy; c Department of Soil, Plant and Food Sciences, University of Bari, Italy

## Abstract

This data article contains mineralogical and chemical data of the wulfenite (PbMoO_4_) sampled from mine of Fiumarella in Calabria region (Southern Italy). Wulfenite is a rare mineral belonging to the class of minerals called molybdates and if found in large amounts it can be used for the extraction of molybdenum. In the mine of Fiumarella, in addition to primary minerals such as barite, galena, cerussite, anglesite, fluorite and chalcopyrite, wulfenite was also detected. Wulfenite crystals are bipyramidal, few microns in size and grow as single crystals that can reach 1 mm. Methods for obtaining the data sets include optical microscopy, micro X-Ray Fluorescence and micro-Raman spectroscopy.

**Specifications Table**

**Table:** 

Subject area	*Earth science*
More specific subject area	*Mineralogy*
Type of data	*Tables, figures*
*How data was acquired*	*Stereo binocular (Askania, GSZ 2T, Germany) fitted with a digital camera (Fuji X-E2, Japan).*
	*Elemental semiquantitative analysis of the mineral were done using an M4 Tornado (Bruker Nano GmbH, Berlin, Germany) µXRF spectrometer equipped with a Rh tube with a policapillary optic (50 kV, 600 μA, 30 W, 25 μm of spot size) and with two XFlash® silicon drift detectors having 30 mm^2^ of active area and a resolution lower than 140 eV (Mn-Kα). Analysis were led under vacuum (20 mbar) and 120 s of live time were chosen. Finally, only one detector was used and the second one was employed to detect ambiguous diffraction peaks. Spectrum deconvolution and quantification were performed using the M4 Tornado analysis software.*
	*Micro-Raman analyses were performed using a Thermo Fisher DXR Raman microscope (Waltham, MA, USA), equipped with OMNICxi Raman Imaging software 1.0, an objective of 50×, a grating of 900 ln/mm (full width at half maximum, FWHM), and an electron multiplying charge-coupled device (EMCCD). The 532.0-nm line (solid state laser) was used at an incident power output at 6 mW. The spatial resolution of the laser beam was about 3–5 µm.*
Data format	*Data collection and Analysis*
Experimental factors	*Samples were investigated non destructively without any modification*
Experimental features	*Crystals were recovered under optical microscope; micro X-Ray Fluorescence and micro-Raman spectrometer without pretreatment.*
Data source location	*38°55׳19.19" N, 16°34׳25.71" E, Fiumarella mine, Catanzaro province, Calabria Region, Italy*
*Data accessibility*	*Data is with this article*

**Value of the data**

• For the first time, wulfenite crystals have been detected and characterized from Calabria region (Italy) for both local and global comparisons.
• The data presented will allow to enrich the scientific knowledge of the wulfenite of Fiumarella mine (Catanzaro - Italy) for a possible extraction of molybdenum.
• The data set can be used to valorize the location of Fiumarella, according to the Convention on the Protection of World, Cultural and Natural Heritage, adopted by UNESCO in 1972.
• The data presented here may be used by other authors to compare composition, morphological features and Micro-Raman bands of other wulfenite crystals discovered in other parts of the world.
• The data can be compared with those obtained from similar geologic environments and motivate studies on rare oxide minerals in the future.

## 1 Data

**Fig. 1:**
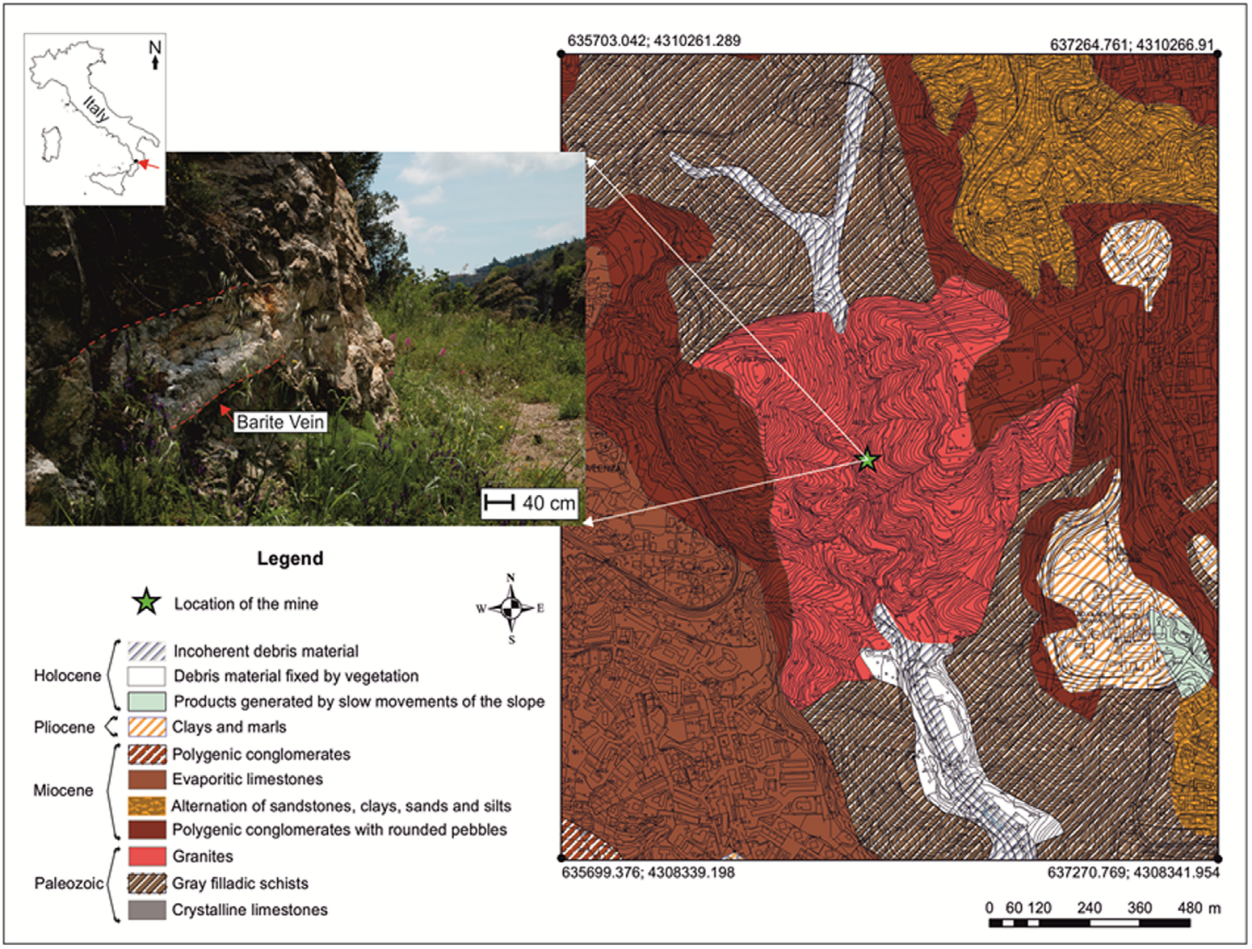
Location of the Fiumarella mine and geological map of the area. The barite vein where the wulfenite crystals were found is also shown.

**Fig. 2:**
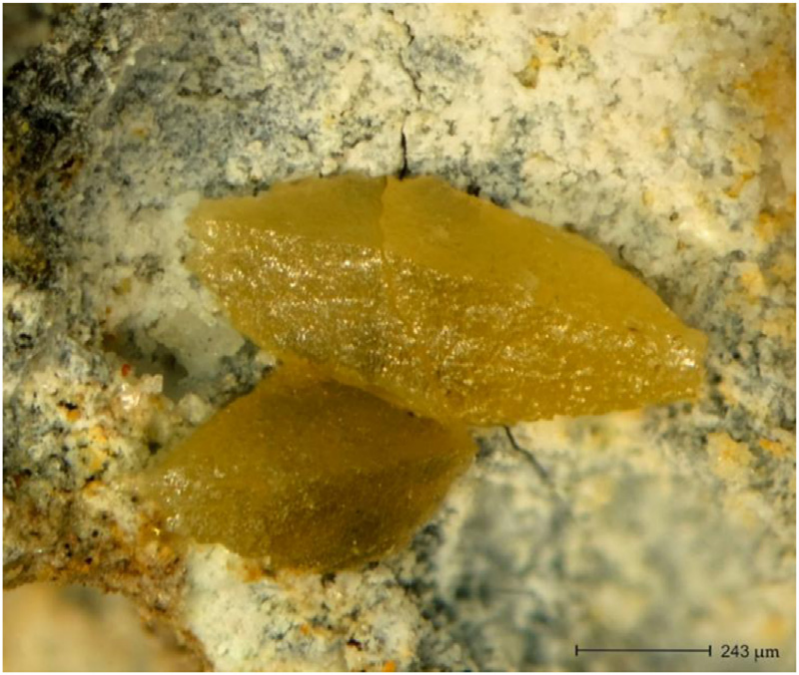
Optical image of yellow-honey wulfenite protruding from matrix.

**Fig. 3:**
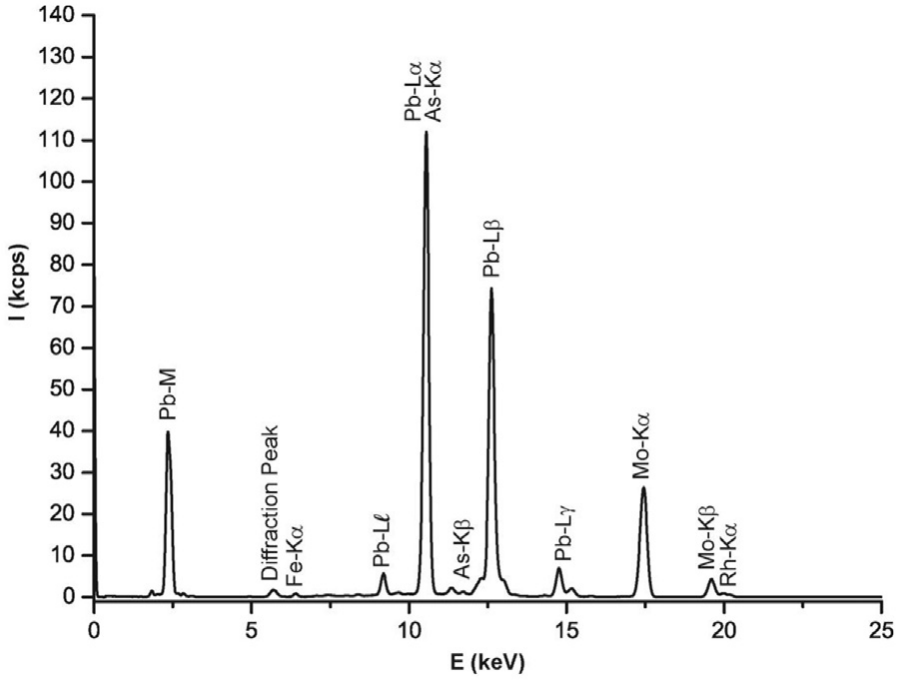
Micro X-ray fluorescence (µXRF) spectrum of the wulfenite mineral coming from the Fiumarella mine (Catanzaro - Southern Italy).

**Fig. 4:**
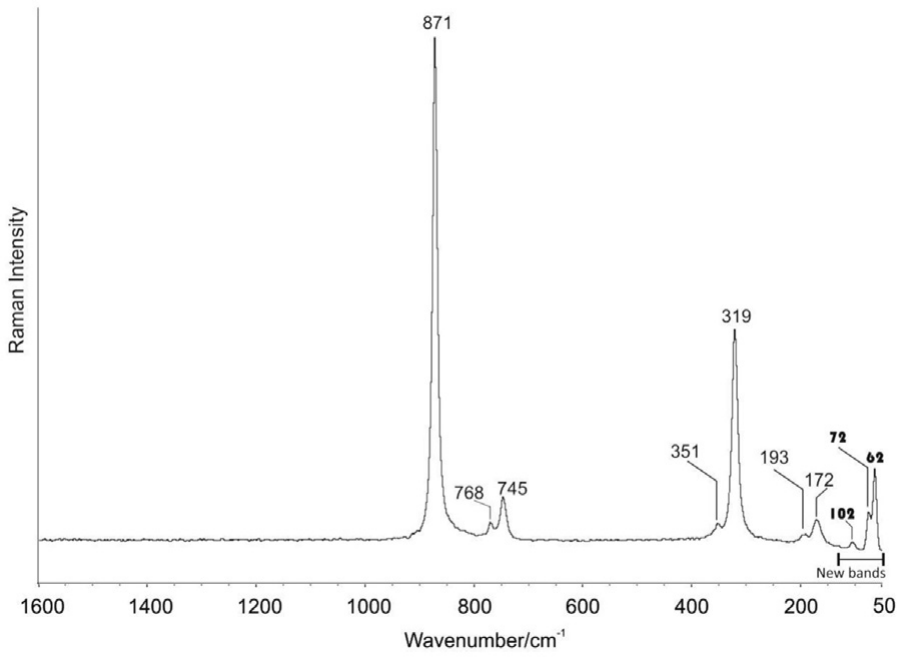
Raman spectra of the wulfenite mineral coming from the Fiumarella mine (Catanzaro - Southern Italy). The bands indicated as bold text have never been published in previous works.

This data article contains mineralogical and chemical data of wulfenite (PbMoO_4_) sampled from the barite mine of Fiumarella in Calabria Region (Southern Italy) (Fig. 1). The crystals of wulfenite were identified and characterized by optical microscopy, micro-Raman spectroscopy and micro X-Ray Fluorescence. Wulfenite occurs as a secondary mineral in the oxidation zones of hydrothermal lead deposits [1], [2], [3]. Wulfenite colour and morphology are shown in Fig. 2. A representative chemical analysis of wulfenite crystals is given in Fig. 3. The chemical composition of wulfenite as for the major constituents and some impurity elements is shown in Table 1. The characteristic Raman spectrum of the wulfenite and the its typical bands are shown respectively in Fig. 4 and Table 2.

**Table 1:** Semi-quantitative composition of wulfenite as determined by µXRF. Oxide concentrations (Conc.) and standard deviation (SD) are expressed in weight percentage (wt.%).

Oxide	Conc.	SD
PbO_2_	61.38	0.28
MoO_3_	37.81	0.08
Fe_2_O_3_	0.18	0.01
As_2_O_3_	0.63	0.01

**Table 2:** Micro-Raman Band wavenumber/cm^−1^ for the wulfenite coming from Fiumarella mine (Catanzaro - Southern Italy).

Mineral׳s name	Bands wavenumber/cm^−1^ according with references [6], [7], [8] and RRUFF database	New bands wavenumber/cm^−1^ identified in this study
Wulfenite	172, 193, 319, 351, 745, 768 and 871 cm^−1^	62, 72 and 102 cm^−1^

## 2 Experimental design, materials and methods

### 2.1 Study area description

The Fiumarella mine (38°55'19.19" N, 16°34'25.71" E) is located in the western part of Catanzaro town, along Fiumarella Creek, in location Molino Mastricarro (Fig. 1). The area has been intensively exploited till the end of the eighties and about 5000 m of galleries have been excavated mainly to extract barite, fluorite, galena and chalcopyrite [1], [2], [3].

### 2.2 Optical microscopy

Specimens were investigated using a stereo binocular (Askania, GSZ 2 T, Germany) and images of the wulfenite crystals were acquired using a digital camera (Fuji X-E2, Japan). All the wulfenite crystals are yellow-honey in color with a greasy to resinous luster (Fig. 2) and appear to be idiomorphic forming bipyramidal tetragonal crystals. This morphology, similar to that found in the Austrian Bleiberg deposit [4], has been rarely detected worldwide. Based on the observation of several crystals, the bipyramid has a length to width ratio of about 3.5 to 1.5. Wulfenite crystals occur as overgrowth on a matrix containing white-grey, granular massive cerussite.

### 2.3 Micro X-Ray fluorescence (µXRF) data

Elemental semiquantitative analysis of the mineral were done using an M4 Tornado (Bruker Nano GmbH, Berlin, Germany). As regards chemical composition, the major elements include Pb and Mo (most intense peaks in the µXRF spectrum), chemically consistent with wulfenite composition (Fig. 3). In this regard, it is worth remembering that wulfenite is a mineral of broad economic importance due to its rarity and the extraction of molybdenum [5]. The semi-quantitative chemical composition of ten crystals as determined by µXRF are reported in Table 1. Note that the determined chemical composition deviates only slightly from the ideal formula PbMoO_4_. Specifically, the PbO_2_/MoO_3_ ratio corresponds to 1.62, indicating a lower value than the theoretical value of 1.66 probably for the following reasons: i) µXRF chemical data are acknowledged to be semi-quantitative; ii) low amounts of Fe and As present as impurities deriving from the matrix are also detected (Table 1).

### 2.4 Micro-Raman spectroscopy data

Micro-Raman analyses were performed using a Thermo Fisher DXR Raman microscope (Waltham, MA, USA). Fig. 4 shows the Raman spectrum of the wulfenite crystals with typical bands at 172, 193, 319, 351, 745, 768 and 871 cm^−1^ [6], [7], [8]; these data (Table 2) are in agreement with the spectrum present in the RRUFF database named “R050024”. The bands at 62, 72 and 102 cm^−1^ (Fig. 4 and Table 2) also belong to wulfenite and they have been detected for the first time in this study.
